# K_V_3.1 channel modulation: a systematic review of pharmacological intervention strategies

**DOI:** 10.3389/fphar.2026.1821275

**Published:** 2026-07-31

**Authors:** Eider Nuñez, Arantza Muguruza-Montero, Ane Arrizabalaga-Iriondo, Julian Zayas-Arrabal, Sara M-Alicante, Janire Urrutia, Miren Revuelta

**Affiliations:** 1 Department of Physiology, Faculty of Medicine and Nursing, University of the Basque Country (UPV/EHU), Leioa, Bizkaia, Spain; 2 Instituto Biofisika CSIC–EHU, Leioa, Spain; 3 Biobizkaia Health Research Institute, Pediatric Oncology, Barakaldo, Spain; 4 Biobizkaia Health Research Institute, Otolaryngology and Language and Communication Disorders, Barakaldo, Spain

**Keywords:** electrophysiology, inhibitors, KCNC1, Kv3.1 channel, pharmacology, positive allosteric modulators, therapeutic modulation

## Abstract

**Introduction:**

Voltage-gated potassium channels of the K_V_3 subfamily, particularly K_V_3.1 (encoded by KCNC1), are essential regulators of fast-spiking inhibitory interneuron activity and high-frequency neuronal firing, enabling precise control of neuronal excitability and network synchrony. Growing evidence links K_V_3.1 dysfunction to epilepsy, schizophrenia, tinnitus, fragile X syndrome, amyotrophic lateral sclerosis, and KCNC1 related developmental and epileptic encephalopathies, positioning this channel as a promising therapeutic target.

**Methods:**

This systematic review, conducted in accordance with PRISMA guidelines, evaluates original studies published between 2016 and January 2026 that investigated pharmacological modulation of K_V_3.1 using *in vitro*, *in vivo*, structural, and translational approaches.

**Results:**

A total of thirty-two studies met the predefined PICOS criteria. The literature reveals two pharmacological strategies: positive allosteric modulation aimed at enhancing fast-spiking inhibitory interneuron function and restoring excitation inhibition balance, and state-dependent channel inhibition, particularly relevant for pathogenic gain of function KCNC1 variants.

**Discussion:**

While early positive allosteric modulators demonstrated proof of mechanism with limited clinical success, second-generation compounds exhibit improved translational potential, including evidence that they modulate functional brain networks in humans. In parallel, clinically approved antidepressants have been identified as open-channel blockers of K_V_3.1, enabling mutation-specific therapeutic repurposing.

**Conclusions:**

Collectively, these findings highlight K_V_3.1 modulation as a context-dependent and increasingly precise pharmacological strategy for neurological and neurodevelopmental disorders**.**

## 1 Introduction

Ion channels are essential to cellular physiology as they control electrical excitability and signal transduction (Dai, 2023). Within ion channels group, potassium (K^+^) channels constitute one of the largest and most diverse subgroups, playing a crucial role in neuronal signaling and muscle excitability (Maljevic and Lerche, 2013).

Based on their biophysical and regulatory properties, K^+^ channels are divided into four major families: inwardly rectifying (Kir) (Hibino et al., 2010), two-pore domain (K2P) (Schreiber and Seebohm, 2026), calcium-activated (KCa) (Latorre et al 1989; Orfali and Albanyan, 2023) and voltage-gated (K_V_) potassium channels (Taura et al., 2021).

### 1.1 Voltage-gated potassium channels (K_V_)

The K_V_ channel family represents the most diverse group of K^+^ channels and is fundamentally defined by its sensitivity to membrane potential (Kim and Nimigean, 2016). These channels contain a specialized voltage-sensing domain, typically the S4 segment that detects membrane depolarization and triggers channel opening in response to electrical stimuli. This activation facilitates K^+^ efflux, driving membrane repolarization. As the primary mediators of the falling phase of the action potential (AP), K_V_ channels play a central role in shaping AP duration, regulating firing frequency, and controlling overall cellular excitability in excitable cells such as neurons (Kim and Nimigean, 2016).

**FIGURE 1:**
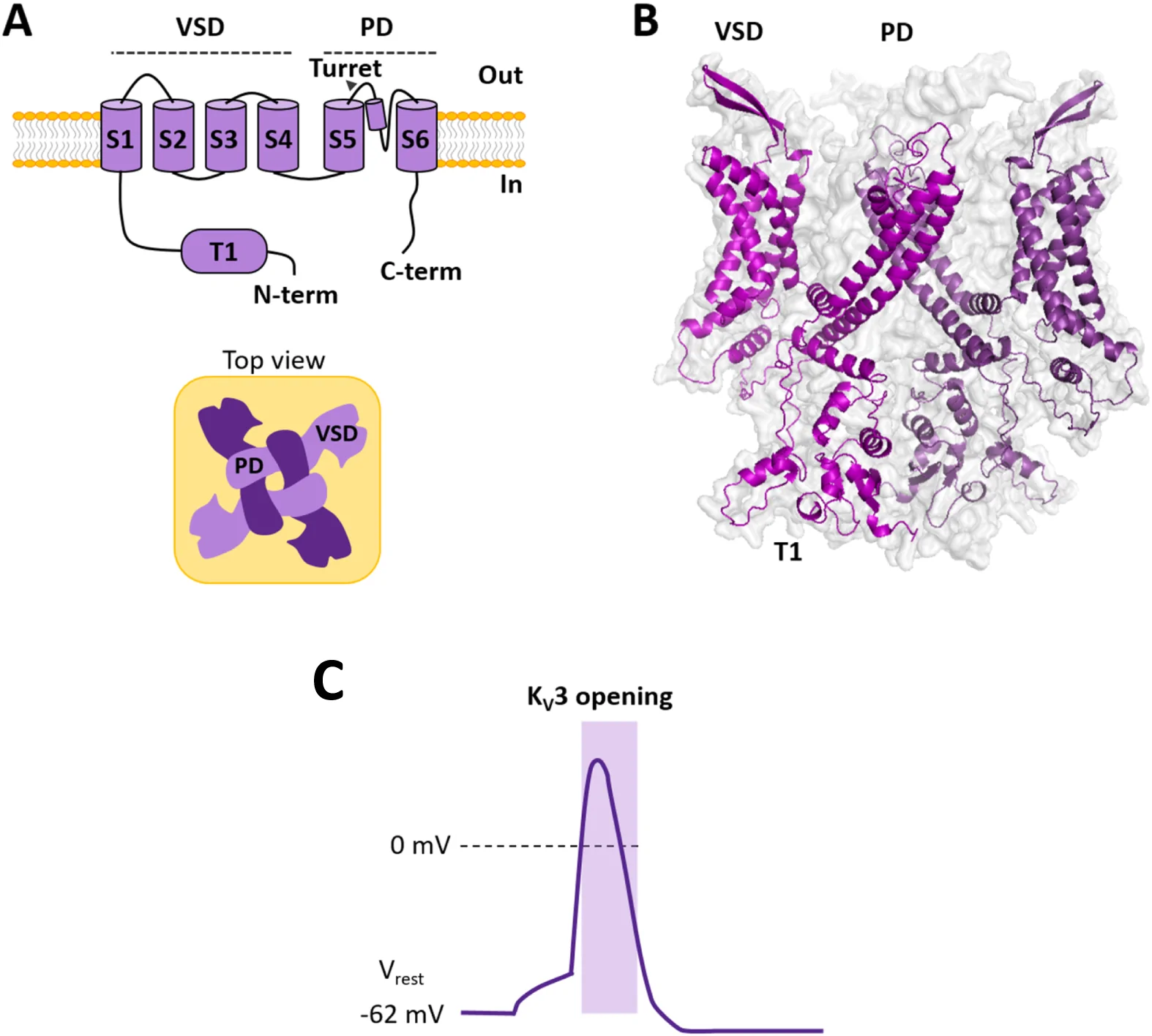
General properties of K_v_3 family. **(A)** Schematic representation of K_v_3 subunit. VSD, voltage-sensing domain; PD, pore domain; T1, tetramerization domain. The top view shows the assembly of four subunits necessary to form the functional channel. **(B)** AlphaFold structure prediction of K_v_3.1 subunit. Only two subunits are shown. **(C)** K_v_3 channels follow fast-gating kinetics, opening at a relatively depolarized membrane potential. For general electrophysiological recordings of the K_v_3 family members, see Kaczmarek and Zhang (2017).

Structurally, K_V_ channels have a tetrameric architecture (Figures 1A,B), with each subunit containing six transmembrane helices (S1-S6). The S1-S4 segments form the voltage sensor domain (VSD), while the S5-S6 segments constitute the pore domain, including the extracellular turret connecting S5 to the pore loop, which can influence channel gating and interactions with extracellular ligands (McCoy and Nimigean, 2012; Liang et al., 2024). The cytoplasmic N- and C-terminal domains of K_V_ channels act as modulatory regions, influencing channel gating and function (Barros et al., 2012).

These channels, encoded by approximately 40 genes, are classified into 12 subfamilies (K_V_1-K_V_12) based on genetic and structural homology (Wulff et al., 2009). Each subfamily exhibits distinct tissue-specific expression profiles. Notably K_V_1, K_V_2, K_V_3, K_V_4, K_V_7 are predominantly expressed in excitable cells, like neurons and cardiomyocytes and their functional properties are further diversified by electrically silent or modulatory subunits, including K_V_5, K_V_6, and K_V_9 (Kilfoil et al., 2019; Chow and Leung, 2020; Musselman et al., 2023).

#### 1.1.1 K_V_3 subfamily

Among the K_V_ subfamilies, K_V_3 channel subfamily comprises 4 members: K_V_3.1, K_V_3.2, K_V_3.3 and K_V_3.4. Those channels are distinguished by their unique biophysical and functional characteristics (Kaczmarek and Zhang, 2017). Their fast-gating kinetics and activation at relatively depolarized membrane potentials make them key mediators of rapid AP repolarization in the nervous system (Covarrubias et al., 2025). These properties enable neurons to sustain high-frequency firing, thereby supporting the fast-spiking phenotype of inhibitory interneurons, cerebellar neurons and auditory brainstem neurons, contributing to the precise timing of neuronal signaling (Rudy and McBain, 2001; Kaczmarek and Zhang, 2017).

Structurally, K_V_3.1 (encoded by KCNC1) resembles other K_V_ channels structure with a distinctive feature in the configuration of their cytoplasmic tetramerization domain (T1), which provides a structural basis for precise control of channel gating (Chi et al., 2022) (Figures 1A,B).

#### 1.1.2 K_V_3.1 channels as therapeutic targets

Dysregulation in K_V_3.1 channels has been associated with several neurological and auditory disorders (Li et al., 2021). These observations have motivated the development of selective K_V_3.1 modulators, which can enhance or suppress channel activity to restore proper neuronal excitability.

To date, several pharmacological modulators have been investigated for their capacity to selectively target K_V_3.1 channels as potential therapeutic agents. K_V_3.1 channels are subject to bi-directional modulation to regulate neuronal excitability. Positive modulators, such as AUT1, AUT2, and AUT00206, shift the voltage-dependent activation toward more hyperpolarized potentials, thereby increasing current availability and enhancing high-frequency firing fidelity (Brown et al., 2016; Kaar et al., 2022). This mechanism suggests therapeutic potential for treating epilepsy and schizophrenia. Conversely, negative modulators and blockers like Paroxetine reduce outward current and slow deactivation, resulting in broader action potentials and increased refractory periods (Erisir et al., 1999; Rudy and McBain, 2001). These pharmacological tools are essential for defining the role of K_V_3.1 in fast-spiking inhibitory interneuron dysfunction and rhythmic circuit oscillations.

This review provides a systematic overview of the most recent literature on pharmacological agents targeting KV3.1 channels. Throughout the manuscript, the term “fast-spiking interneurons” refers to inhibitory GABAergic interneurons, predominantly parvalbumin-positive cells, as commonly described in the cerebral cortex, hippocampus, and striatum, unless otherwise specified in the original studies.

## 2 Materials and methods

This systematic review was structured and reported in accordance with the Preferred Reporting Items for Systematic Reviews and Meta-Analyses (PRISMA) guidelines. The framework comprises a 27-item checklist, the PRISMA abstract checklist, and a flowchart diagram (Page et al., 2021).

### 2.1 Search strategy

A comprehensive literature search was conducted using the following online databases: Pubmed/Medline, Web of Science (WoS), Scopus, and Cochrane library. The search strategy employed the following terms: “K_V_3.1” or “KCNC1” combined with “therapeutic modulation” or “drug” or “modulator”. Detailed search strings for each database, ensuring full reproducibility of the search, are provided in Table 1.

**TABLE 1:** Search items used in Pubmed, Scopus, WOS and Cochrane library.

Database	Search strategy
Pubmed	(Kv3.1 OR KCNC1) AND (modulator OR drug) NOT (REVIEW [Publication type]); English; full text (Kv3.1 OR KCNC1) AND (therapeutic modulator) NOT (REVIEW [Publication type])
Scopus	((TITLE-ABS-KEY ((kv3.1 OR KCNC1)) AND TITLE-ABS-KEY ((modulator OR drug))) AND PUBYEAR >2015 AND PUBYEAR <2026 AND (LIMIT-TO (DOCTYPE,“ar”)) ((TITLE-ABS-KEY ((kv3.1 OR KCNC1)) AND TITLE-ABS-KEY ((therapeutic modulator))) AND PUBYEAR >2015 AND PUBYEAR <2026 AND (LIMIT-TO (DOCTYPE,“ar”)))
Web of science (WOS)	(“Kv3.1″OR “KCNC1” OR “KCNC1 gene”) AND (modulat* OR activat* OR inhibit* OR block* OR “positive allosteric modulator”
Cochrane library	“Kv3.1” in all text OR “KCNC1” in all text AND “modulator” in publication type AND English in language NOT “review” in publication type - (word variations have been searched) “Kv3.1” in all text OR “KCNC1” in all text AND “drug” in publication type AND English in language NOT “review” in publication type - (word variations have been searched) “Kv3.1” in all text OR “KCNC1” in all text AND “therapeutic modulator” in publication type AND English in language NOT “review” in publication type - (word variations have been searched)

### 2.2 Eligibility criteria

The Population, Intervention, Comparison, Outcomes, and Study (PICOS) framework was employed to guide the selection of articles (Methley et al., 2014). The *population* comprised all animal and cell models; the *intervention* encompassed studies examining the relationship between any pharmacological treatment and K_V_3.1 potassium channels. The *comparison* was made against control groups; the *outcome* assessed was the effect of the drug in K_V_3.1 channel functionality; and finally, the study design included all *in vivo*, *in vitro,* or structural analysis. Specific criteria regarding publication timeframe (2016–2026) and article type (excluding reviews) were applied. The publication timeframe (2016–2026) was defined *a priori* to focus on recent advances in K_V_3.1 pharmacology, particularly those with translational and clinical relevance. While this approach may exclude earlier seminal studies, key foundational contributions have been incorporated to provide historical and mechanistic context.

Filters were applied to restrict the search according to these predefined eligibility criteria. While these filters substantially reduced the number of retrieved records, a small number of non-eligible papers remained and were subsequently removed manually during the screening process. All titles and abstracts were screened to identify and exclude duplicates.

The following exclusion criteria were applied prior to constructing the PRISMA flowchart:

- Studies not published in English.
- Studies published prior to 2016.
- Review articles.
- Studies investigating potassium channels other than K_V_3.1.
- Articles for which full text is inaccessible.

### 2.3 Study selection

Following the initial database search and removal of duplicated articles, title and abstract screening was conducted by two independent researchers (AM-M and EN) to exclude irrelevant articles, as well as those that did not meet the predefined inclusion criteria. Subsequently, the full texts of the remaining potentially relevant articles were carefully evaluated by two researchers (JU, AA-I) to assess their suitability based on methodology and results. Any discrepancies between the researchers were resolved through consultation with other researchers (MR, JZ). Subsequently, three independent researchers (AA-I, JU, EN) extracted the following information from the selected studies: compound, targets, mechanistic description, clinical indication, developmental stage and author. Discrepancies were resolved through a consensus-based approach with a third reviewer until 100% agreement was reached.

### 2.4 Risk of bias assessment

To ensure the robustness of our findings, risk of bias was assessed across three domains using tailored frameworks: *in vitro* studies were evaluated using an adapted version of the NTP-OHAT-adapted (National Toxicology Program Office of Health Assessment and Translation) tool, focusing on key quality parameters including controls, blinding, independent replicates, cell authentication, and dose-response patterns; *in vivo* studies were scrutinized employing the Systematic Review Centre for Laboratory Animal Experimentation risk of bias tool (SYRCLE tool), prioritizing randomization, baseline consistency, blinding, and the handling of outliers or selective reporting; and structural biology evidence was validated through the Assessment of Structural Inference Validity, centering on construct, environment, fitting, dynamics, and computational reproducibility and final structural resolution (Table 2). Two independent reviewers (MR and AA-I) assessed the following domains, selection bias, performance bias, detection bias, and reporting bias. Discrepancies were resolved by a third reviewer (AM-M).

**TABLE 2:** Risk of bias evaluation.

Author	Model	ROB	Item 1	Item 2	Item 3	Item 4	Item 5	Overall rating
Anderson et al. (2018)	*In vitro*	ADAPTED BASED ON NTP-OHAT	+	+	+	+	-	4
Boddum et al. (2017)			+	?	+	+	+	4
Martins et al. (2016)			+	?	+	+	+	4
Lee et al. (2023)			+	?	+	+	+	4
Lee et al. (2018)			+	?	+	+	+	4
Munch et al. (2018)			+	?	+	+	-	3
Filareto et al. (2025)			+	?	+	+	?	3
An et al. (2026)			+	-	+	+	+	4
Bassetto Junior et al. (2019)			+	?	+	+	+	4
Bassetto Junior et al. (2017)			+	?	+	+	+	4
Ambrosino et al. (2023)			+	?	?	+	+	3
El-hassar et al. (2019)			+	+	+	+	-	3
Chambers et al. (2017)			+	+	+	+	-	3
Chen et al. (2023)			+	+	+	+	+	4
Maatoug et al. (2021)			+	?	?	+	+	3
Brown et al. (2016)			+	?	+	+	+	4
Liang et al. (2024)			+	+	+	+	-	4
Rybalko et al. (2021)	*In vivo*	SYRCLE	+	+	-	+	-	3
Glait et al. (2018)			-	+	+	+	+	4
Kaar et al. (2024)			+	+	?	?	+	3
Sanchez et al. (2020)			+	?	+	+	?	3
El-hassar et al. (2019)			-	+	-	+	+	3
Chambers et al. (2017)			+	+	-	?	+	3
Thelin et al. (2017)			-	+	+	?	+	3
Parekh et al. (2018)			?	+	-	+	+	3
Kaar et al. (2022)			+	+	?	?	+	3
Angelescu et al. (2022)			+	+	?	?	+	3
Feng et al. (2024)			?	?	?	+	+	3
Marabita et al. (2025)			+	+	?	?	+	3
Andrade-Talaver y. (2020)			+	+	?	+	+	4
Carlyon et al. (2018)			+	+	+	-	+	4
Lauber et al. (2016)			+	+	-	-	+	3
Chambers et al. (2017)	Structural biology	Assessment of structural inference validity	+	+	+	+	?	4
Thelin et al. (2017)			+	?	+	-	+	3
Chen et al. (2023)			+	+	?	+	?	3
Liang et al. (2024)			+	+	+	+	?	4
Botte et al. (2022)			+	+	+	+	+	5

Scoring system (adapted from NTP-OHAT for *in vitro* studies and SYRCLE for *in vivo* studies): Each item (e.g., blinding, randomisation, independent replicates) was rated as: “+” = low risk of bias (criterion clearly met), “?” = unclear risk (insufficient information), “–” = high risk of bias (criterion not met or clearly flawed). The overall rating (0–5) is a qualitative summary: 0–1 = very high risk; 2 = high risk; 3 = moderate risk; 4 = low risk; 5 = very low risk. This rating reflects the number of items with low risk and the absence of critical flaws. For example, a score of 4 indicates that most items were rated “+” with one or two “?” but no “–”; a score of 3 indicates at least one ‘–’ or several “?” that could affect confidence.

Studies with an overall risk-of-bias rating of ≤2 (high or very high risk) were excluded. However, no study met this exclusion threshold, as all included studies received a rating of ≥3 (moderate to very low risk). Thus, risk of bias was not used as an exclusion criterion in practice, but only to qualitatively appraise the strength of the evidence and to guide interpretation of findings in the Discussion.

## 3 Results

### 3.1 Selection of articles

**FIGURE 2:**
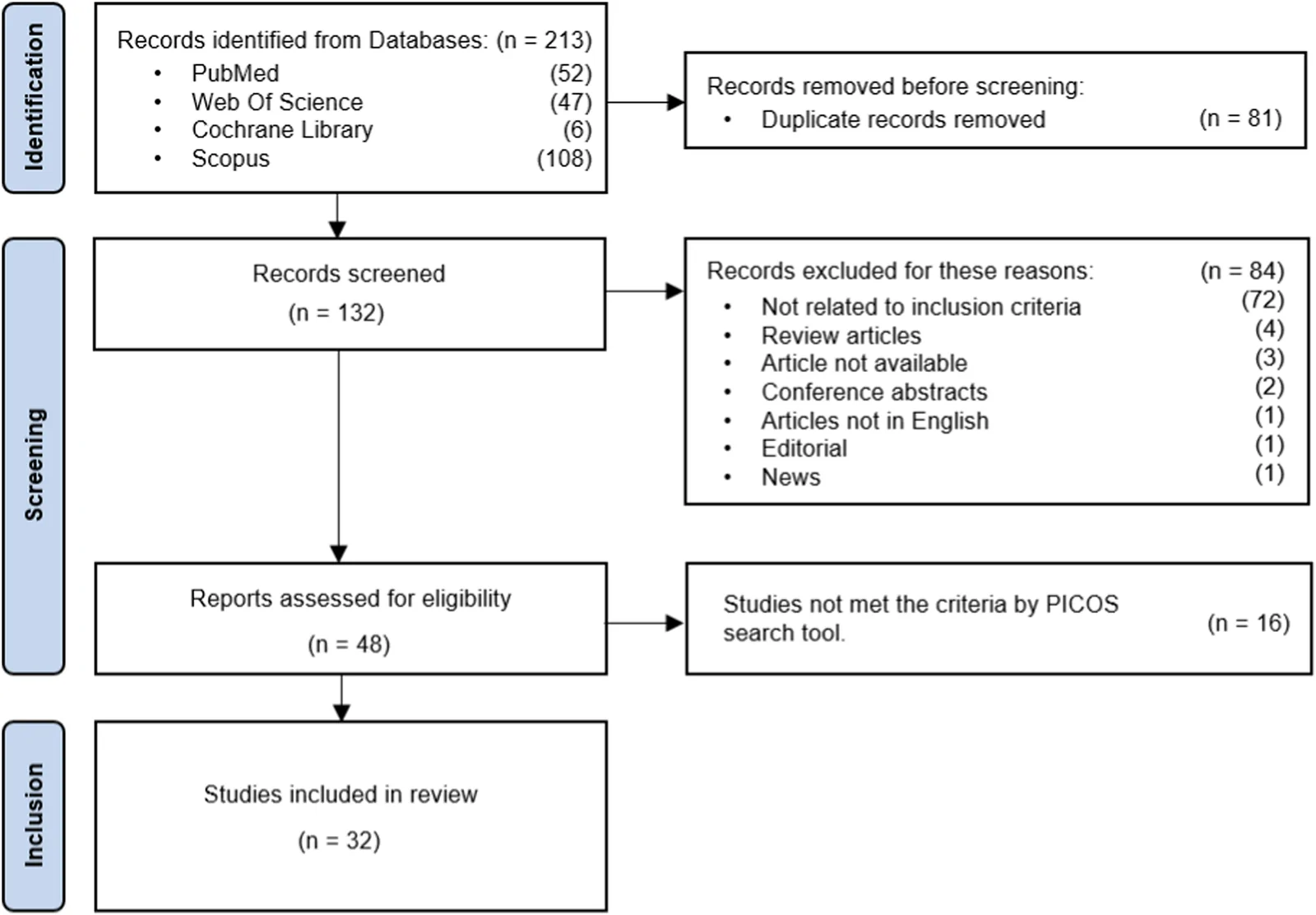
PRISMA flowchart diagram. Workflow. Of the 213 articles that were found, 32 were ultimately analyzed, as they met the established criteria.

A total of 213 articles were identified across all databases: PubMed (52), Web of Science (47), Cochrane Library (6), and Scopus (108). Duplicate records were removed using Microsoft Excel (n = 81), yielding 132 articles for screening. Based on the predefined inclusion criteria, further 84 records were excluded as described in Figure 2. The remaining 48 studies were assessed for eligibility, of which 32 were ultimately included in the review. 16 studies were excluded as they did not meet the PICOS criteria. The complete study selection process is illustrated in the PRISMA flow diagram (Figure 2).

### 3.2 Characteristics of the included studies

The studies included in this systematic review examined pharmacological modulation of K_V_3.1 channels using a broad range of experimental approaches, including molecular, electrophysiological, and clinical analysis. The risk of bias assessment (Table 2) revealed that included studies (n = 32) demonstrated high methodological quality, with overall ratings ranging from 3 to 5. Overall, the literature can be categorized into two major pharmacological strategies: positive allosteric modulation and channel inhibition, with both approaches explored in preclinical models and, in a subset of cases, in clinical or translational contexts.

Across the included studies, considerable heterogeneity was observed in experimental design, disease models, outcome measures, and compound potency and selectivity.

Nevertheless, the overall body of evidence consistently supports K_V_3.1 channels as druggable targets whose modulation impacts fast-spiking neuronal activity, network synchrony, and disease-relevant phenotypes. These shared characteristics provide the framework for the subsequent subdivision of results into K_V_3.1 activators (positive allosteric modulators) and K_V_3.1 inhibitors, which are discussed in detail in the following sections.

### 3.3 Positive allosteric modulators of K_V_3 channels

The studies included in this systematic review focused on positive allosteric modulators (PAMs) of K_V_3 channels, particularly K_V_3.1 (KCNC1), reflecting a strong interest in enhancing fast-spiking neuronal activity and restoring excitation-inhibition balance in neurological and neuropsychiatric disorders. These compounds were characterized across multiple experimental systems, including heterologous expression systems, neuronal cell culture, animal models, and, in selected cases, clinical and translational studies. Overall, PAMs consistently shifted the voltage-dependent activation toward more hyperpolarized potentials, slowed channel deactivation, increased current amplitude, and improved the fidelity and regularity of high-frequency firing in fast-spiking neurons.

#### 3.3.1 AUT00063: first-generation K_V_3 PAM and translational limitations

AUT00063 was the first K_V_3.1/K_V_3.2-selective PAM to be extensively characterized (Chambers et al., 2017; Anderson et al., 2018). In heterologous expression systems, AUT00063 increased K_V_3.1a, K_V_3.1b, and K_V_3.2 currents in a concentration-dependent manner (pEC_50_ ≈ 5.1 ± 0.17 at −15 mV, 7.9 μM), with weaker effects on K_V_3.3 and K_V_3.4 (Anderson et al., 2018). Biophysically, AUT00063 enhanced current amplitude at depolarized potentials and slowed channel deactivation, thereby prolonging K_V_3-mediated repolarizing currents (Anderson et al., 2018).

In fusiform neurons (FNs) of the dorsal cochlear nucleus (DCN) application of 30 μM of AUT00063 reduced the variability of action potential timing and increased the negativity of the hyperpolarization phase. Moreover, Chambers et al. (2017) identified a critical threshold at which neurons transition to a highly reliable response pattern, where each stimulus evokes a single AP tightly phase-locked to the stimulus onset. That threshold was reached when approximately 48% of standard K_V_3.1 conductance was modulated by the compound. However, it is worth noting that the experimental concentration of 30 μM required to elicit these responses in brain slices substantially exceeds the compound’s (7.9 μM). This discrepancy underscores a critical pharmacological limitation, as such high working concentrations may compromise subtype selectivity and trigger off-target effects in complex neuronal networks.

K_V_3.1 channels play a critical role in the auditory brainstem by enabling fast-spiking neurons to sustain high-frequency and temporally precise firing, which is essential for sound localization and temporal processing (Kaczmarek and Zhang, 2017). In particular, neurons in the dorsal cochlear nucleus and inferior colliculus rely on K_V_3-mediated currents to maintain precise spike timing. Disruption of this temporal fidelity has been linked to auditory processing deficits and tinnitus-related hyperactivity (Anderson et al., 2018; Glait et al., 2018).

Preclinical research has focused on the compound’s ability to restore auditory function and temporal processing. Auditory brainstem response (ABR) studies in rats and mice demonstrated that the compound does not affect hearing sensitivity, as no changes in ABR thresholds were observed in young or aged rats, even at high doses of 60 mg/kg (Rybalko et al., 2021). Regarding signal processing, at this same high dose, the compound significantly improved gap prepulse inhibition (gap-PPI), across all tested latencies, suggesting an enhancement of the ability to detect short silent gaps. Moreover, in noise-exposed mice and hamsters, AUT00063 attenuated the increase in spontaneous firing of neurons derived from the inferior colliculus (IC) of rats (Anderson et al., 2018). While these findings suggest a potential to modulate the pathological hyperactivity considered a primary correlate of tinnitus (Glait et al., 2018), this effect remains strictly characterized at the preclinical level and must not be directly equated to a reversal of the clinical condition in humans).

Despite robust preclinical efficacy, clinical translation was limited. In patients with subjective tinnitus, a randomized double-blind placebo-controlled trial failed to demonstrate significant benefit, and no improvements were observed in cochlear implant users (Anderson et al., 2018; Hall et al., 2019). These negative results were attributed to disease heterogeneity, suboptimal clinical endpoints, and the relatively weak modulatory potency of AUT00063 in humans (Rybalko et al., 2021).

Although AUT00063 consistently improved temporal precision and reduced hyperexcitability in auditory preclinical models, these effects were highly dependent on acute pharmacological modulation in controlled experimental systems. Importantly, the absence of efficacy in human tinnitus trials highlights the translational gap between electrophysiological normalization and clinically meaningful symptom improvement. Differences in disease heterogeneity, chronicity, and species-specific auditory circuitry may partially explain these discrepancies.

The “translational class” reflects the degree of preclinical-to-clinical maturity of each compound within the K_V_3 modulator discovery program and does not imply direct chemical lineage between compounds. “Later-generation” compounds refer to structurally optimized tool molecules primarily developed for mechanistic and structural investigation (e.g., subtype selectivity and heteromer sensitivity), whereas “second-generation” compounds denote candidates with improved translational and/or clinical profiles relative to first-generation AUT modulators.

#### 3.3.2 AUT1 and AUT2: mechanistic validation in neuropsychiatric and neurodevelopmental models

**FIGURE 3:**
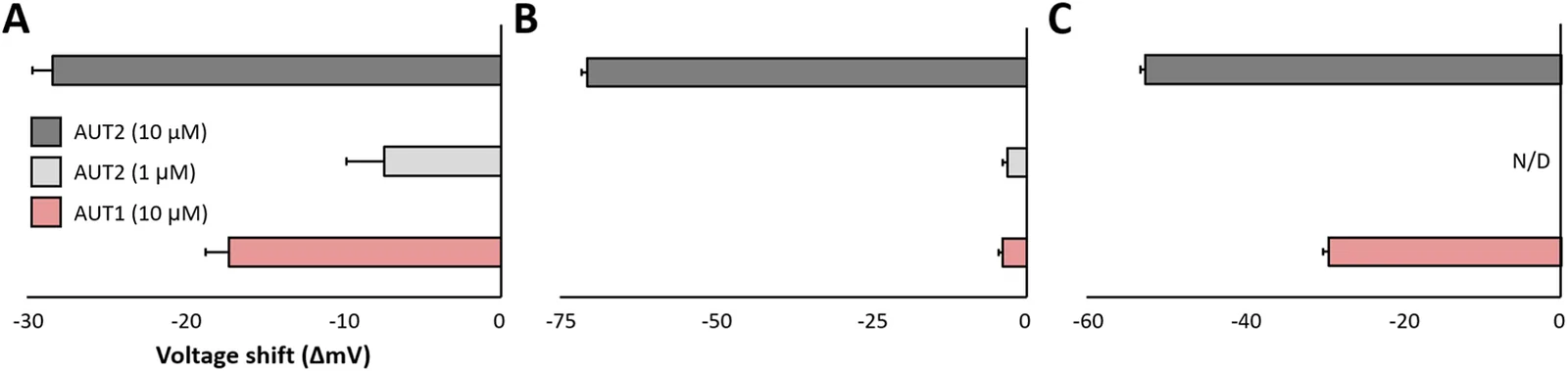
Effects of AUT1 and AUT2 on the shift of voltage-dependent activation and inactivation of K_V_3.1 in CHO cells. **(A)** Both AUT1 and AUT2 shift the V_1/2_ of the voltage-dependent activation of K_V_3.1 toward more hyperpolarized membrane potentials [-17.21 ± 1.46 ΔmV for AUT1 (10 µM); −7.37 ± 2.32 ΔmV for AUT2 (1 µM); −28.35 ± 1.27 ΔmV for AUT2 (10 µM)]. **(B)** The V_1/2_ of the voltage-dependent steady-state inactivation is shifted to more negatives potentials by both AUT1 and AUT2. Nevertheless, only 10 µM of AUT2 produces a significant change in the V_1/2_ [-3.88 ± 0.70 ΔmV for AUT1 (10 µM); −3.13 ± 0.72 ΔmV for AUT2 (1 µM); −70.53 ± 0.95 ΔmV for AUT2 (10 µM)]. **(C)** 10 µM of both AUT1 and AUT two provoke a shift in V_1/2_ of the voltage-dependent cumulative inactivation potential (−29.34 ± 0.77 ΔmV for AUT 1 (10 µM); −52.57 ± 0.58 ΔmV for AUT2 (10 µM)). N/D refers to no data. Data modified from Brown et al. (2016).

AUT1 (also referred to as RE01) and AUT2 are first generation K_V_3.1-selective PAMs and were instrumental in establishing proof-of-mechanism for pharmacological modulation of K_V_3.1 channels *in vivo*. These compounds provided early evidence that enhancing K_V_3.1 function can modulate pathological neuronal firing patterns, translating into behavioral and sensory phenotypes relevant to preclinical models of neuropsychiatric and neurodevelopmental disorders (Brown et al., 2016; Parekh et al., 2018). At the biophysical level, compared with AUT00063, both AUT1 and AUT2 produced larger hyperpolarizing shifts in activation gating and stronger effects on neuronal firing fidelity, suggesting improved pharmacodynamic engagement. These compounds act through a shared mechanism as K_V_3.1-selective positive allosteric modulators, shifting the voltage-dependent activation of K_V_3.1 toward more hyperpolarized membrane potentials (Figure 3A) and thereby increasing channel availability during physiological firing (Brown et al., 2016; Boddum et al., 2017). In heterologous expression systems (CHO cells), AUT1 increased K_V_3.1 current amplitude and slowed channel deactivation without significantly affecting steady-state inactivation or K_V_3.1b expression levels (Figure 3B) (Brown et al., 2016). However, voltage-dependent cumulative inactivation was significantly shifted to more hyperpolarized potentials (−12.7 ± 0.26 vs. −42.04 ± 0.73 mV. Figure 3C). AUT2 exhibited concentration-dependent modulation of gating properties: at a higher concentrations produced a marked leftward shift in half steady-state inactivation voltage, suggesting engagement of additional gating states that may constrain the therapeutic window (Figure 3) (Brown et al., 2016). These findings provided an early quantitative framework for understanding how subtle modulation of K_V_3.1 gating can alter neuronal excitability in a dose-dependent manner.

At the cellular and functional levels, AUT1 demonstrated a cell-type specific modulation of firing properties in midbrain circuits involved in dopaminergic signaling. In dopaminergic neurons, AUT1 (10 μM) broadened action potentials, slowed repolarization, and reduced firing rates during high current injections without affecting spike amplitude, while increasing firing irregularity. In contrast, in GABAergic inhibitory interneurons, AUT1 did not significantly alter spike waveform or mean firing frequency, but increased firing regularity. This pattern is predicted to enhance the temporal precision of inhibition and rebalance excitation/inhibition within mesolimbic networks (Parekh et al., 2018). Consistent with these cellular effects, AUT1 reduced firing rates in auditory brainstem neurons enriched in K_V_3.1 channels, further supporting its preferential impact on fast-spiking neuronal populations (Brown et al., 2016).

These electrophysiological properties translated into robust, genetically validated behavioral outcomes in rodent model. In CD1 mice, prophylactic administration of AUT1 produced a dose-dependent prevention of amphetamine-induced hyperlocomotion, a widely used indicator for hyperdopaminergic states. Importantly, this effect was strictly dependent on functional K_V_3.1 channels, as AUT1 reduced hyperactivity in wild-type and K_V_3.2 knockout mice but was completely ineffective in K_V_3.1 knockout animals, thereby providing strong genetic evidence of on-target engagement (Parekh et al., 2018). In the ClockΔ19 mouse model of mania-like behaviour, which exhibits pathological hyperactivity, acute AUT1 administration selectively normalized locomotor activity without affecting baseline behavior in wild-type controls, indicating a favorable state-dependent effect profile and minimal impact on physiological motor function (Parekh et al., 2018).

In parallel, AUT2 provided mechanistic validation of K_V_3.1 modulation in a neurodevelopmental disease model characterized by sensory hypersensitivity. In fact, AUT2 was primarily investigated in the context of Fragile X syndrome-related auditory processing deficits. In the Fmr1^-/y^ mouse model of Fragile X syndrome, neurons of the medial nucleus of the trapezoid body (MNTB) exhibit an increased spike threshold (44.21 ± 1.37 mV in Fmr1^-/y^ vs. 37.28 ± 1.35 mV in WT) and increased K^+^ currents that activate at relatively positive membrane potentials (i.e., a right-shifted voltage dependence), with the current amplitude at high depolarizations being 5.90 ± 0.40 nA in Fmr1−/− vs. 3.99 ± 0.66 nA in WT. These biophysical alterations contribute to abnormal auditory brainstem responses (ABRs) and auditory hypersensitivity (Brown et al., 2016; El-Hassar et al., 2019). Systemic administration of AUT2 (30 mg/kg) is proposed to act by shifting the voltage-dependent activation of K_V_3.1 channels toward more negative potentials, thereby compensating for the pathological right-shift and restoring normal high-frequency firing fidelity in auditory circuits. This mechanism normalized ABR wave IV amplitude and reduced auditory hypersensitivity *in vivo*, without simply reducing the overall current amplitude. Notably, the efficacy of AUT2 depended on endogenous K_V_3.1 expression levels, which vary across tonotopic gradients in the auditory brainstem and are altered in disease states, highlighting a major source of inter-regional and inter-individual variability in responsiveness to K_V_3.1 PAMs (Rosato-Siri et al., 2015; Brown et al., 2016).

Overall, the strength of evidence varied substantially across compounds. While some PAMs, such as AUT00063 and AUT00206, progressed to translational or early clinical evaluation, many other compounds remain limited to mechanistic or structural studies in heterologous systems. Consequently, conclusions regarding therapeutic efficacy should be interpreted according to the level of experimental validation available for each compound.

#### 3.3.3 AUT5: subtype-selective modulation and structural determinants

AUT5 (also described as EX15; see Autifony, https://autifony.com/science/) was identified as a highly selective K_V_3.1/K_V_3.2 PAM with minimal activity on other K_V_3 family members (Liang et al., 2024) and represents a later-generation compound within the AUT development pipeline.

Functional characterization across multiple experimental systems, including *Xenopus laevis* oocytes expressing recombinant K_V_3 subunits, HEK293 cells, and acute mouse brain slices, showed that it accelerates K_V_3 activation kinetics, shifts voltage-dependent activation toward more hyperpolarized potentials, and enhances high-frequency neuronal firing, particularly in K_V_3.1 and K_V_3.2 channels (Andrade-Talavera et al., 2020). In hippocampal neurons, AUT5 enhanced the ability of fast-spiking interneurons to follow high-frequency stimulation, supporting its role in modulating neuronal excitability in circuits requiring precise temporal firing (Andrade-Talavera et al., 2020).

Cryo-electron microscopy (Cryo-EM) structural analyses, combined with two-electrode voltage clamp (TEVC) recordings in *Xenopus* oocytes, revealed that AUT5 shares a common binding region with earlier PAMs such as AUT1. Structural studies indicate that this selectivity arises from interactions with the extracellular turret domain, a structural feature not conserved in other K_V_ channel families (e.g., K_V_1, K_V_2, K_V_7) (Liang et al., 2024). Moreover, Liang et al. (2024) found that the assembly of K_V_3.1/K_V_3.4 heteromultimers, which occur naturally in certain neurons, alters the pharmacological response to AUT5. In particular, K_V_3.4 subunits exhibit N-type fast inactivation, which can significantly influence the net effect of modulators on channel kinetics and neuronal excitability. These findings underscore the importance of channel stoichiometry and molecular context in predicting therapeutic responses to K_V_3 PAMs.

Notably, additional pharmacological profiling revealed a more complex behavior, as AUT5 was also reported to inhibit currents mediated by K_V_3.2 and K_V_3.4 channels under certain conditions, indicating subtype- and context-dependent effects (Boddum et al., 2017). This dual profile highlights the need for careful evaluation of net effects on neuronal excitability and reinforces the importance of comprehensive subtype characterization when developing K_V_3-targeting compounds.

#### 3.3.4 AUT00206 and AUT00201: second-generation PAMs with *in vivo* and translational relevance

AUT00206 and AUT00201 represent more potent, second-generation K_V_3 PAMs developed to overcome the limited efficacy, selectivity, and clinical translation challenges of first-generation AUT compounds. While no direct medicinal chemistry lineage has been publicly disclosed, these second-generation molecules were rationally designed within the same K_V_3-targeting program to improve target engagement, pharmacokinetic properties, and *in vivo* efficacy relative to earlier AUT series compounds (Feng et al., 2024; Marabita et al., 2025).

In a mouse model of progressive myoclonus epilepsy type 7 (EPM7), AUT00206 partially restored impaired fast-spiking properties of K_V_3-expressing neurons, which in mutant animals exhibit prolonged afterhyperpolarization and reduced firing frequencies, consistent with compromised high-frequency firing capacity (Feng et al., 2024). AUT00206 modestly increased K_V_3 channel conductance and shifted the voltage-dependent activation toward more hyperpolarized potentials in both mutant and wild-type channels, indicating preservation of its positive allosteric mechanism across pathological and physiological contexts. At the cellular level, AUT00206 enhanced repetitive firing in cerebellar granule cells and improved spike fidelity in parvalbumin-positive interneurons during high-frequency stimulation, two neuronal populations critically dependent on K_V_3 channel function for temporal precision. Systemic administration of AUT00206 reduced seizure frequency and severity and significantly improved motor coordination *in vivo*, providing direct evidence that K_V_3 potentiation can rescue circuit dysfunction and behavioral deficits in a neurodevelopmental epilepsy context (Feng et al., 2024).

Consistent with translational relevance, AUT00206 also demonstrated measurable effects on human brain circuits implicated in schizophrenia. Functional neuroimaging and EEG studies revealed modulation of striatal and cortical network activity, including reductions in frontal gamma power (Kaar et al., 2024), a biomarker associated with excitation-inhibition imbalance and the severity of positive psychotic symptoms (Baradits et al., 2019). Importantly, positron emission tomography (PET) imaging indicated that the clinical and neurophysiological effects of AUT00206 were not accompanied by changes in dopamine synthesis capacity, supporting a mechanism of action driven by direct modulation of cortical and striatal network dynamics rather than classical dopaminergic pathways (Angelescu et al., 2022; Kaar et al., 2022). Together, these findings suggest that K_V_3 PAMs may engage circuit-level phenotypes relevant to humans disease. However, current evidence remains limited to early translational biomarkers, and further clinical studies are required to determine whether these neurophysiological effects translate into meaningful therapeutic benefit.

AUT00201 further expanded the therapeutic scope of K_V_3 modulation by exhibiting broader activity across K_V_3 channel subtypes. In automated patch-clamp assays, AUT00201 increased human K_V_3.1, K_V_3.2, and K_V_3.4 currents measured at −15 mV in a concentration-dependent manner, with pEC_50_ values of 6.15 ± 0.04 (K_V_3.1), 6.52 ± 0.05 (K_V_3.2), and 5.60 ± 0.04 (K_V_3.4), demonstrating differential potency across subtypes while maintaining functional activity on channels expressed in both neuronal and non-neuronal populations. In the SOD1-G93A mouse model of amyotrophic lateral sclerosis (ALS), chronic AUT00201 treatment attenuated motor dysfunction, reduced motor neuron loss, and ameliorated muscle pathology (Marabita et al., 2025). Although neuromuscular junction pathology was not significantly modified, treatment was associated with a ∼1.3-fold increase in the number of surviving motor neurons in the ventral spinal cord compared with vehicle-treated controls, indicating a neuroprotective effect. Moreover, AUT00201 significantly attenuated microglial and astrocytic activation in the spinal cord, suggesting that K_V_3 modulation may influence not only intrinsic neuronal excitability but also neuroinflammatory components of disease progression (Marabita et al., 2025).

AUT00206 compound is currently undergoing phase I clinical trials to evaluate its safety and tolerability, while AUT00201 has advanced to Phase IIb to assess its efficacy and optimal dosing in patient populations.

#### 3.3.5 Additional K_V_3 PAMs

Several additional K_V_3 channel modulators, and a series of small molecules referred to as Compounds 1–4, have been described as PAMs with heterogeneous binding sites and context-dependent mechanisms of action (Chen et al., 2023). This compounds share a conserved structural mechanism of action with the classic imidazolidinedione-based AUT compound series (such as AUT1 and AUT5) (Chen et al., 2023; Liang et al., 2024). These small molecules generally promote K_V_3 channel opening at more hyperpolarized membrane potentials, decelerate deactivation kinetics, and enhance high-frequency neuronal firing, thereby increasing the capacity of fast-spiking interneurons to sustain rapid and temporally precise firing patterns, a mechanism highly relevant for reversing cognitive deficits in schizophrenia (Chen et al., 2023). Structural, mutagenesis, and *in silico* docking studies have identified extracellular domains and inter-domain interfaces, including regions within the turret and voltage-sensor and pore domain coupling interfaces, as critical determinants of ligand binding and subtype selectivity, underscoring the structural plasticity of K_V_3 channels as drug targets (Chen et al., 2023).

#### 3.3.6 LuAG00563: a structurally defined K_V_3.1 PAM

In addition to the AUT series compounds, LuAG00563 has been identified as a positive allosteric modulator of K_V_3.1 channels through high-resolution structural studies (Botte et al., 2022). Using cryo-electron microscopy (cryo-EM), LuAG00563 was found to bind to a novel site within the transmembrane domain of K_V_3.1, distinct from the binding sites of previously described modulators such as AUT1 or AUT5. This interaction stabilizes the activated/open state of the channel, thereby enhancing K_V_3.1-mediated currents. Although LuAG00563 has been used primarily as a preclinical tool compound to validate structural mechanisms, its detailed characterization provides a rational basis for future design of more selective and efficacious K_V_3 modulators. Therefore, this compound should be explicitly mentioned alongside other PAMs in the pharmacological landscape of K_V_3.1 channels.

Despite consistent electrophysiological effects across studies, interpretation of the overall efficacy of K_V_3 PAMs is limited by substantial methodological heterogeneity, including differences in expression systems, species, disease models, dosing paradigms, and electrophysiological endpoints. Moreover, most evidence derives from acute preclinical experiments, whereas long-term effects, compensatory mechanisms, and safety profiles remain incompletely characterized.

### 3.4 Inhibitors of K_V_3.1 channels: open-channel blockers and repurposed drugs

Although most drug discovery efforts targeting K_V_3.1 channels have focused on PAMs, growing evidence highlights the therapeutic and translational relevance of K_V_3.1 inhibitors, many of which act as state-dependent open-channel blockers (Lee et al., 2018; 2023; Bassetto Junior et al., 2019). These compounds originate from diverse sources, including clinically approved antidepressants and metabolic drugs, synthetic small molecules, and natural toxins. Despite their structural heterogeneity, a unifying feature among many K_V_3.1 inhibitors is their preference for the open channel state, allowing access to binding sites within or near the ion-conducting pore (Lee et al., 2018; Bassetto Junior et al., 2019; Maatoug et al., 2021).

#### 3.4.1 Open-pore block of K_V_3.1 channels by antidepressants

A particularly important and initially unexpected class of K_V_3.1 inhibitors comprises selective serotonin reuptake inhibitors (SSRIs) and related antidepressants. Over the past decade, multiple first-line antidepressants have been demonstrated to act as state-dependent open-channel blockers of K_V_3.1 channels, producing concentration-dependent inhibition of macroscopic currents and altering channel gating kinetics (Lee et al., 2018; Ambrosino et al., 2023; Filareto et al., 2025). This mechanism represents a well-characterized off-target effect with context-dependent clinical relevance. While K_V_3.1 inhibition occurs at micromolar concentrations *in vitro*, which are typically above those achieved under standard antidepressant dosing, it provides a mechanistic framework for understanding neuropsychiatric manifestations observed under supratherapeutic conditions, including overdose (Lee et al., 2018; An et al., 2026). In addition, these findings have stimulated interest in drug repurposing strategies in precision medicine, particularly in the context of gain-of-function potassium channel mutations associated with neuronal hyperexcitability disorders (Ambrosino et al., 2023; Filareto et al., 2025).

Electrophysiological characterization of K_V_3.1 channel inhibition by antidepressant drugs reveals a shared pharmacological mechanism among selective serotonin reuptake inhibitors, notably paroxetine and fluoxetine (Lee et al., 2018; Ambrosino et al., 2023; Filareto et al., 2025), which extends to the serotonin-norepinephrine reuptake inhibitor duloxetine (An et al., 2026). Whole-cell patch-clamp recordings in heterologous expression systems demonstrate that these compounds act as state-dependent open-channel blockers (Lee et al., 2018; Filareto et al., 2025; An et al., 2026). These agents share a characteristic electrophysiological signature: concentration-dependent reduction of macroscopic current amplitude, accelerated apparent current decay during depolarization, use-dependent enhancement of block, and marked slowing of channel deactivation. The latter frequently manifests as a tail-current crossover phenomenon, consistent with drug trapping within the open inner pore cavity (Lee et al., 2018; Filareto et al., 2025; An et al., 2026). Paroxetine exhibits voltage-dependent inhibition with a fractional electrical distance of approximately 0.5, indicating that the drug senses roughly half of the transmembrane electric field as it binds from the intracellular side of the pore (Lee et al., 2018). Despite structural differences among these antidepressants, their convergent electrophysiological and kinetic profiles strongly support a shared binding determinant or overlapping interaction site within the K_V_3.1 inner cavity.

Quantitative analyses reveal notable differences in inhibitory potency across compounds. In CHO cells expressing cloned rat K_V_3.1b channels, paroxetine inhibits K_V_3.1 currents with an IC_50_ of 9.43 µM (Lee et al., 2018), whereas fluoxetine displays a lower potency, with an IC_50_ of approximately 17.0 µM for wild-type channels (Ambrosino et al., 2023). Duloxetine emerges as the most potent inhibitor, exhibiting a steady-state IC_50_ of 2.04 µM in recombinant systems (An et al., 2026). Importantly, these findings extend beyond heterologous expression models. Duloxetine produces a comparable degree of K_V_3.1 inhibition in human neuroblastoma SH-SY5Y cells, which endogenously express K_V_3.1 channels, yielding an IC_50_ of ∼1.98 µM (An et al., 2026). For fluoxetine, a critical translational advance lies in its efficacy against the gain-of-function K_V_3.1-V425M variant associated with KCNC1-related developmental and epileptic encephalopathy. Fluoxetine blocks currents generated by this mutant with an IC_50_ of 8.7 µM, providing a direct basis for its therapeutic benefit in this context (Ambrosino et al., 2023). This *in vitro* finding was successfully translated in a patient carrying the K_V_3.1-V425M variant (Filareto et al., 2025). Treatment with fluoxetine (0.26 mg/kg/day) resulted in complete seizure control, allowed the withdrawal of valproate, and led to marked behavioral improvement and reduced epileptic activity on EEG over more than 5.5 years of follow-up.

Structural insights are most advanced for duloxetine, where molecular docking studies predict high-affinity interactions at two distinct sites: one within the central ion-conducting pore and another at the intracellular interface between the voltage-sensing and pore domains, consistent with its state-dependent blocking behavior (An et al., 2026).

The clinical interpretation of these data requires careful consideration of drug exposure. Under standard antidepressant dosing, plasma concentrations of these agents, exemplified by duloxetine levels of approximately 0.1–0.4 µM, remain well below the concentrations required for substantial K_V_3.1 inhibition, typically by a factor of 5–50 (An et al., 2026). As a result, direct K_V_3.1 blockade is unlikely to contribute meaningfully to therapeutic efficacy in depression or anxiety. However, this off-target activity becomes clinically relevant under specific conditions. In overdose, plasma drug levels may approach the range necessary to inhibit K_V_3.1 channels, offering a plausible mechanistic explanation for severe neuropsychiatric adverse effects such as confusion and hallucinations, which may arise from impaired firing of fast-spiking interneurons (Lee et al., 2018; An et al., 2026). The reported use of fluoxetine in a single patient, with KCNC1-related developmental and epileptic encephalopathy, to suppress seizures and improve neurological outcomes suggests that K_V_3.1 blockade may represent a potentially useful therapeutic strategy in selected gain-of-function variants (Ambrosino et al., 2023).

While the clinical success of fluoxetine in a single patient with the K3.1−V425M variant is a promising proof-of-concept for precision medicine, these findings must be interpreted with extreme caution. The evidence remains limited to isolated clinical cases, and large-scale validation is required to confirm the safety and efficacy of antidepressant repurposing in this population.

#### 3.4.2 Synthetic compounds, natural toxins, and other clinically approved inhibitors

Beyond antidepressants, a heterogeneous group of K_V_3.1 inhibitors has been described, including synthetic small molecules, venom-derived peptides, and FDA-approved drugs. Many of these compounds share a use-dependent, open-channel blocking mechanism, despite marked structural diversity (Bassetto Junior et al., 2019).

Among synthetic inhibitors, benzenesulfonamide derivatives have received particular attention. Several members of this family inhibit K_V_3.1 channels, including SMD3, SMD2_APP, SMD3_APP, SMD2_SN, and SMD3_SN (Martins et al., 2016; Bassetto Junior et al., 2019). SMD2 (4-chloro-3-nitro-N-butylbenzenesulfonamide) emerges as the most potent compound, acting as a reversible, use-dependent blocker that binds to an extracellular site on the membrane. SMD2 inhibits K_V_3.1 with an IC_50_ of 9.3 ± 0.6 µM and is currently in preclinical studies as a potential anticonvulsant candidate (Bassetto Junior et al., 2017).

Natural toxins provide high-affinity molecular probes for K_V_3.1. The scorpion venom-derived peptide α-KTx 15.1, isolated from *Androctonus australis hector*, has been proposed to inhibit K_V_3.1 *via* direct pore occlusion, with Lys27 interacting with the selectivity filter region according to docking studies. Inhibition in the nanomolar range (∼170 nM) has been reported; however, these values were obtained using an eight-peptide mixture from a purified venom fraction rather than the isolated peptide. While this limits quantitative interpretation, α-KTx 15.1 remains a valuable tool for probing K_V_3.1 pore architecture and gating, although its application is currently restricted to preclinical research (Maatoug et al., 2021).

In addition to α-KTx peptides, earlier studies identified peptide toxins such as blood depressing substance I (BDS-I) and BDS-II, isolated from *Anemonia sulcata*, as potent modulators of K_V_3 channels. These toxins act primarily as gating modifiers, altering channel activation kinetics by shifting the voltage dependence of activation and slowing channel opening, thereby reducing K_V_3-mediated currents (Diochot et al., 1998; Yeung et al., 2005). Despite their relatively high potency, BDS toxins display limited selectivity and significant off-target effects on other potassium channel subtypes, which has restricted their use in pharmacological and translational contexts. Nevertheless, they remain historically important as some of the first compounds demonstrating that K_V_3 channel function can be modulated through gating mechanisms rather than direct pore block, providing an early conceptual framework for the development of modern state-dependent and allosteric modulators.

Several clinically approved drugs also exhibit secondary inhibitory effects on K_V_3.1. Rosiglitazone, an antidiabetic thiazolidinedione, rapidly and reversibly inhibits K_V_3.1-mediated currents with an IC_50_ of ∼29.8 µM, likely through open- or inactivated-state block (Lee et al., 2023). Although its affinity is lower than that of SMD2 or α-KTx 15.1, its FDA approval and commercial availability, make it attractive for repurposing in K_V_3.1-related disorders such as epilepsy and ataxia. However, K_V_3.1 inhibition may impair the fast-spiking capacity of inhibitory interneurons, raising concerns about potential pro-convulsant effects. Therefore, rosiglitazone-based strategies would be most rational for specific gain-of-function KCNC1 mutations.

Valproic acid (VPA), a widely used treatment for epilepsy and psychiatric conditions such as bipolar disorders and acute mania (Haddad et al., 2009) modulating K_V_3.1 through an indirect mechanism involving transcriptional regulation rather than acute channel blockade. Prenatal exposure to VPA, commonly used as an experimental model of autism spectrum disorder, leads to a ∼40% reduction in Kcnc1 (K_V_3.1b) mRNA and protein levels in the forebrain, altering the excitation/inhibition balance (Lauber et al., 2016). Although prenatal VPA exposure is associated with adverse neurodevelopmental outcomes, its ability to downregulate K_V_3.1 expression may have mechanistic relevance when considering therapeutic strategies aimed at counteracting gain-of-function K_V_3.1 variants.

Inhibitor studies also exhibit marked variability in experimental systems and pharmacological characterization. Importantly, many compounds inhibit K_V_3.1 only at micromolar concentrations, raising uncertainty regarding physiological relevance under standard clinical exposure conditions.

## 4 Discussion

**FIGURE 4:**
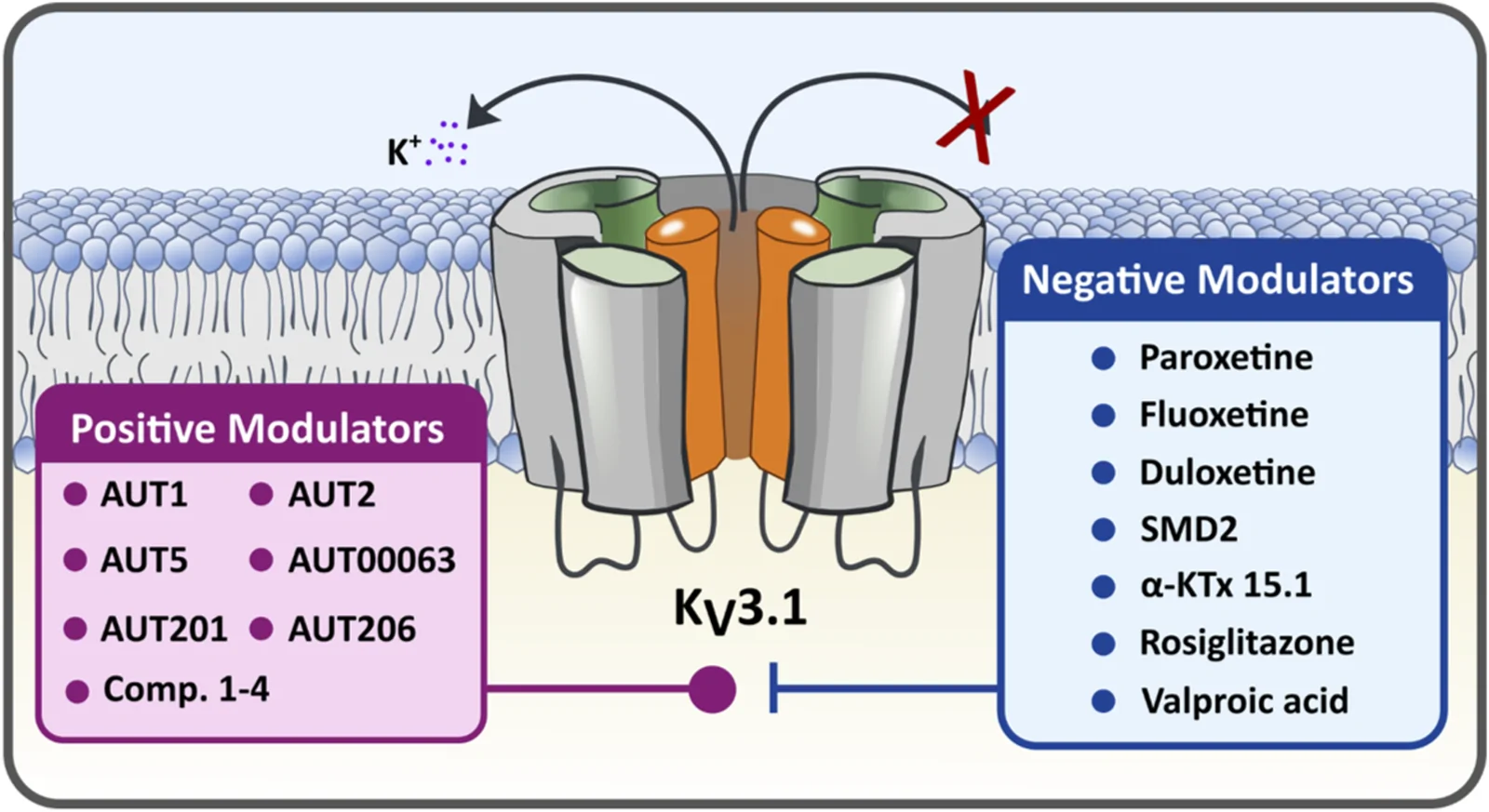
Schematic representation of positive and negative modulators of K_V_3.1.

The pharmacological enhancement of K_V_3.1 and K_V_3.2 channels *via* PAMs represents a promising therapeutic strategy to address the excitation-inhibition imbalances related to several neurological disorders (Figure 4). As demonstrated by first-generation compounds AUT00063 (Chambers et al., 2017; Anderson et al., 2018) and the AUT1/2 series (Brown et al., 2016; Parekh et al., 2018), the underlying biophysical mechanism, shifting the voltage-dependent activation toward hyperpolarized potentials, effectively restores the high-frequency firing fidelity of fast-spiking interneurons. However, the limited clinical success of early candidates like AUT00063 in tinnitus trials (Rybalko et al., 2021) highlights a significant translational gap. This discrepancy reflects disease heterogeneity and the complex stoichiometry of native channels, which can differ significantly from the homomeric expression systems used in early drug discovery.

Recent advancements with second-generation PAMs, specifically AUT00206 and AUT00201, provide a clearer path forward. AUT00206 has demonstrated engagement of human cortical and striatal circuits, modulating biomarkers like gamma-band oscillations without the side effects associated with classical dopaminergic pathways (Kaar et al., 2022; 2024). Furthermore, its ability to rescue circuit dysfunction in epilepsy models (Feng et al., 2024) reinforces therapeutic versatility of this scaffold. Simultaneously, the neuroprotective effects of AUT00201 in ALS models (Marabita et al., 2025) suggest that K_V_3 modulation may extend beyond immediate symptomatic relief to influence long-term neuronal survival and neuroinflammatory responses. The consistency of the pharmacological effects observed across different compounds is supported by the overall low risk of bias in the primary literature, as detailed in our systematic assessment (Table 2).

Looking ahead, the next-generation of K_V_3 therapeutics must navigate the “context-selectivity” revealed by tool compounds like EX15 (Andrade-Talavera et al., 2020) and AUT5 (Liang et al., 2024). Because the assembly of heteromultimers (e.g., K_V_3.1/K_V_3.4) and variations in the extracellular turret can dampen or even invert the effects of a PAM, future research should prioritize stoichiometry-selective drug design. By tailoring modulators to the specific subunit compositions present in pathological states, it may be possible to achieve a more precise, state-dependent normalization of neuronal activity. However, a major limitation of the current evidence base remains the translational gap between simplified recombinant expression systems and the heterogeneous stoichiometry of native K_V_3 channel complexes in the human brain. Furthermore, while the methodological quality of the studies is generally high (Table 2), the reliance on different experimental endpoints, ranging from cellular firing to behavioral assays, makes it difficult to establish a universal scale of efficacy across compounds. Nevertheless, as our understanding of the structural determinants and circuit-level impacts of K_V_3 modulation grows, these PAMs may emerge as essential tools in the precision medicine toolkit for neurodevelopmental and neurodegenerative diseases.

While the search for K_V_3 modulators has long been dominated by the pursuit of channel openers, emerging evidence suggests a potential, yet still limited, therapeutic role of K_V_3.1 inhibitors in specific context (Figure 4). A diverse array of compounds, from synthetic sulfonamides like SMD2 (Bassetto Junior et al., 2017) to the scorpion toxin α-KTx 15.1 (Maatoug et al., 2021), converge on a shared mechanism of state-dependent open-channel blockade. This preference for the open state allows these molecules to access binding sites within the ion-conducting pore, a feature that is particularly relevant for treating gain-of-function (GoF) mutations where the channel remains pathologically active.

One of the most notable translational observation in this field is that several clinically approved antidepressants, including fluoxetine, paroxetine, and duloxetine, have been shown to inhibit K_V_3.1 channels *in vitro* (Ambrosino et al., 2023; Lee et al., 2018). Quantitative evidence indicates that therapeutic plasma concentrations achieved during depression (0.1–0.4 µM) are substantially lower than those required for K_V_3.1 inhibition *in vitro*. As such, direct channel blockade is unlikely to contribute to their clinical effects under standard conditions. However, this pharmacologycal property may become relevant under specific circumstances, such as supratherapeutic exposure or in the presence of gain-of-function (GoF) variants. In this context, the reported use of fluoxetine in a patient carrying the KCNC1-V425M GoF mutation provides a proof-of-principle for precision medicine approaches, although the current evidence remains limited and case-based (Filareto et al., 2025).

Furthermore, other FDA-approved agents like rosiglitazone (Lee et al., 2023) and the transcriptional effects of valproic acid (Lauber et al., 2016) offer alternative routes for modulating K_V_3.1 density and function. However, a major hurdle for future development is the risk of pro-convulsant effects; because K_V_3.1 is essential for the fast-spiking capacity of inhibitory interneurons, non-selective blockade could inadvertently trigger hyperexcitability.

Future efforts should prioritize mutation-specific pharmacology and structure-guided inhibitor design, with particular emphasis on selectively targeting the kinetic abnormalities of mutant channels while preserving the physiological function of wild-type K_V_3.1 populations. Additionally, the discovery of dual-site binding for compounds like duloxetine, targeting both the pore and the voltage-sensor interface, provides a structural rationale for the development of bifunctional inhibitors with increased potency and reduced off-target toxicity.

From a methodological perspective, the present review focuses on recent advances (2016–2026) to capture the most current pharmacological strategies with translational relevance. While this approach may underrepresent earlier seminal work, key historical contributions, including early toxin-based Kv3 modulators such as BDS peptides, have been incorporated to provide mechanistic context. In addition, the restriction to English-language publications introduces a potential risk of language bias, and the analysis remains dependent on the quality and reporting standards of the included studies. These considerations should be considered when interpreting the findings and highlighting the need for continued integration of historical, preclinical, and translational evidence in future work.

## 5 Conclusion

The pharmacological landscape of K_V_3.1 channels has evolved from basic biophysical probes to clinically relevant therapeutic candidates. This systematic review highlights a central duality in K_V_3 drug discovery: the development of PAMs to rescue impaired fast-spiking interneuron activity in neuropsychiatric disorders, and the strategic repurposing of inhibitors to counteract pathogenic gain-of-function mutations. While second-generation PAMs like AUT00206 demonstrate a sophisticated ability to modulate circuit-level dynamics and cortical oscillations, the successful clinical application of fluoxetine in KCNC1 related encephalopathy provides a compelling proof-of-concept for precision medicine.

Nevertheless, significant challenges remain in achieving subtype and context-specific modulation, particularly given the influence of channel stoichiometry and heteromultimerization on drug efficacy. K_V_3.1 modulators represent a promising, non-dopaminergic strategy for neurological disease, provided that next-generation compounds restore pathological excitability while preserving normal interneuron function and minimizing off-target toxicity. Future efforts must integrate structural biology with patient-specific genomic data to transition from broad-spectrum modulation to precision-targeted therapies.

## Data Availability

The raw data supporting the conclusions of this article will be made available by the authors, without undue reservation.

## Supplementary material

SUPPLEMENTARY FIGURE S1 Chemical structures of representative KV3 channel modulators. Top: Positive allosteric modulators (PAMs), including representative structures from the AUT compound series, sharing a common imidazolidinedione scaffold. Bottom: KV3 channel inhibitors, including antidepressants and synthetic small-molecule blockers such as benzenesulfonamide derivatives (e.g., SMD2). These compounds highlight the structural diversity underlying KV3 channel modulation.
