# Correction: KV3.1 channel modulation: a systematic review of pharmacological intervention strategies

**DOI:** 10.3389/fphar.2026.1960510

**Published:** 2026-08-07

**Authors:** Frontiers Production Office

**Affiliations:** Frontiers Media SA, Lausanne, Switzerland

**Keywords:** electrophysiology, inhibitors, KCNC1, Kv3.1 channel, pharmacology, positive allosteric modulators, therapeutic modulation

There was a mistake in Figure 1 as published. Due to a production error, Figure 1C was not an accurate representation of the kinetics of the Kv3 channels being reviewed in the article. Figure 1C has been removed, and the following has been added to the caption: “For general electrophysiological recordings of the Kv3 family members, see **Kaczmarek and Zhang (2017)**”. The corrected Figure 1 and its caption appear below.

**FIGURE 1:**
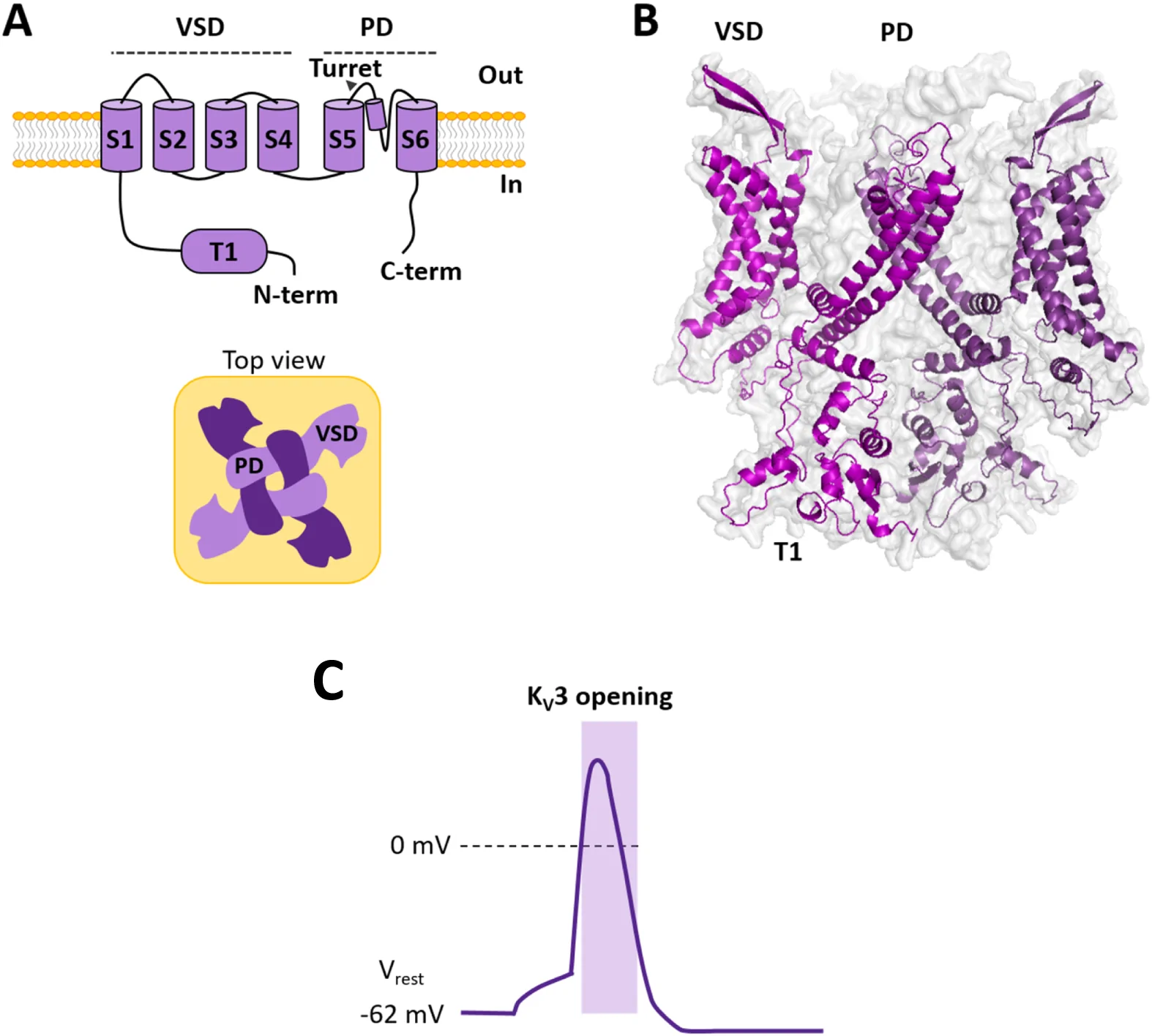
General properties of Kv3 family. **(A)** Schematic representation of Kv3 subunit. VSD, voltage-sensing domain; PD, pore domain; T1, tetramerization domain. The top view shows the assembly of four subunits necessary to form the functional channel. **(B)** AlphaFold structure prediction of Kv3.1 subunit. Only two subunits are shown. **(C)** Kv3 channels follow fast-gating kinetics, opening at a relatively depolarized membrane potential. For general electrophysiological recordings of the Kv3 family members, see Kaczmarek and Zhang (2017).

A correction has also been made to section **1 Introduction**, *1.1.1 KV3 subfamily*, Paragraph 1 to remove the incorrect citation of Figure 1C and replace it with a citation for **Kaczmarek and Zhang (2017)**:

“Among the KV subfamilies, KV3 channel subfamily comprises 4 members: KV3.1, KV3.2, KV3.3 and KV3.4. Those channels are distinguished by their unique biophysical and functional characteristics (**Kaczmarek and Zhang, 2017**).”

The original version of this article has been updated.

